# Supplementary material for: Iron limitation of microbial phosphorus acquisition in the tropical North Atlantic
Source: Nat Commun. 2017 May 19;8:15465. doi: 10.1038/ncomms15465 (PMC5454538; doi:10.1038/ncomms15465)
Supplement: Supplementary Information — Supplementary Discussion, Supplementary Figures, and Supplementary References [file ncomms15465-s1.pdf]

Supplementary Discussion

Computational prediction of the subcellular location of PhoX, PhoD and PhoA from marine bacterial metagenomes suggest a more dominant extracellular or periplasmic localization of PhoX APases, with <20% cytoplasmic localization[1]. In contrast, the same study predicted >40% of PhoA and PhoD marine bacterial APases to be more cytoplasmic in localization[1]. Consequently, in addition to potential involvement in internal cycling[2], some fraction of any in situ PhoA and PhoD activity on extracellular substrates may depend on DOP transported through cell membranes into the cytoplasm[1]. Preferential cytoplasmic localization of PhoA and PhoD could hence potentially complicate interpretation of fluorometric APA assays using substrates that might not be transported across cell membranes (e.g. MUF-P)[1].

There is thus at least the possibility that our experimental design could, under certain conditions, underestimate the potential for Zn limitation of overall DOP hydrolysis rates within the community. Specifically, if Zn was initially limiting the synthesis of cytoplasmic forms of PhoA, it might be expected that MUF-P derived APase responses to subsequent Zn amendment within our experiments may not track the total increase in hydrolysis. Indeed, we note that within some experiments we found APA in NFeZn treated bottles to be lower than that of NFe treated bottles, and in some cases NZn lower than N alone. Although equivocal, one potential explanation for such results could be a shift towards increased reliance on community level P acquisition through enhanced intracellular transport linked to increased cytoplasmic PhoA activity following increased Zn supply. Subsequently, extracellular/periplasmic hydrolysis as measured through the MUF-P APA assay could be hypothesised to be relatively supressed in such circumstances. However, the majority (>50%) of PhoA (and PhoD) APases are still predicted to have periplasimic, outer-membrane or extracellular localizations[1] that would facilitate access to a wide variety of substrates, including MUF-P. Moreover it might also be argued that cytoplasmic forms of APase are likely to play a relatively minor role in overall utilization of the diverse extracellular DOP pool, due to the associated requirement for costly synthesis of a diverse area of compound specific transporters. Ultimately we thus expect our experimental design to be sensitive to both Fe and Zn limitation of DOP hydrolysis, the latter having been demonstrated in previous studies[3]. Moreover, our primary conclusion, that community level APA can be restricted by Fe availability within sub-regions of our study area, is fully robust to any such caveats.

Supplementary Figures

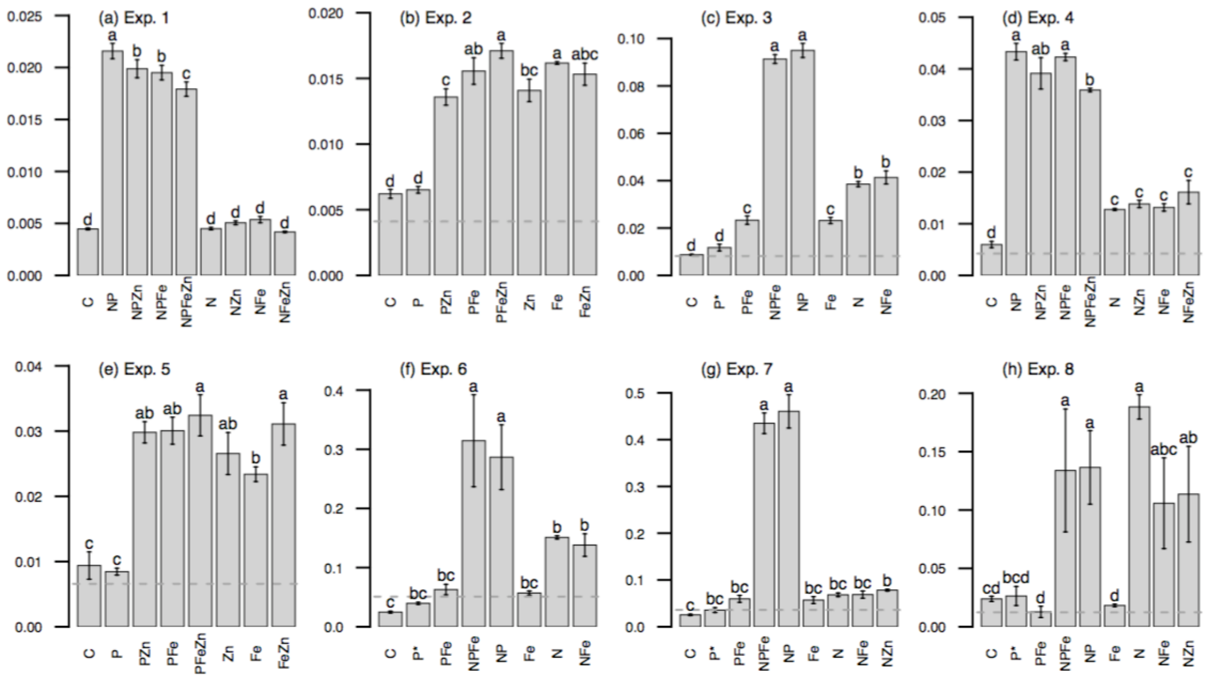

Supplementary Figure 1: *Synechococcus* cell counts. Units: ×10^6 cells mL^{-1}. Shown are means ± standard errors, n=3 for all except where indicated by an asterisk (n=2). The grey horizontal line is the mean initial time point measurement (n=3 apart from Experiment 1, which has no cell counts for the initial time point). Treatment means were compared using a one-way ANOVA and a Fisher PLSD means comparison test (indistinguishable means labelled with the same letter (p<0.05); n.s. is 'not significant').

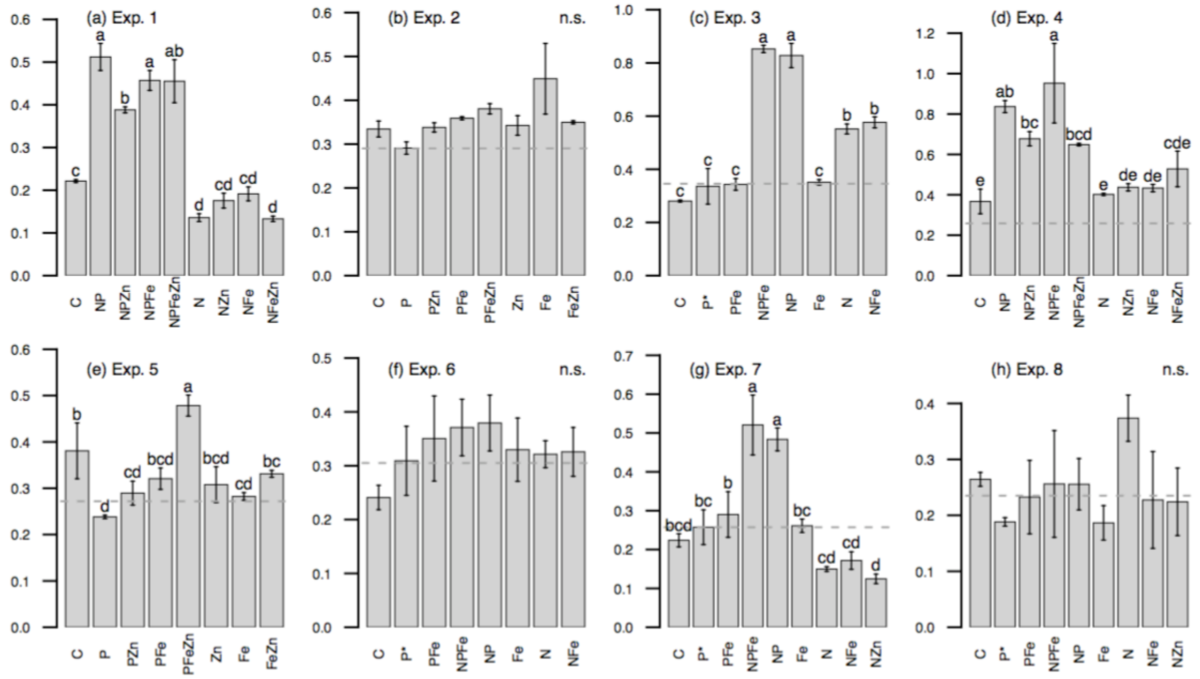

Supplementary Figure 2: *Prochlorococcus* cell counts. Units: $\times 10^6$ cells mL^{-1}. Shown are means ± standard errors, n=3 for all except where indicated by an asterisk (n=2). The grey horizontal line is the mean initial time point measurement (n=3 apart from Experiment 1, which has no cell counts for the initial time point). Treatment means were compared using a one-way ANOVA and a Fisher PLSD means comparison test (indistinguishable means labelled with the same letter (p<0.05); n.s. is 'not significant').

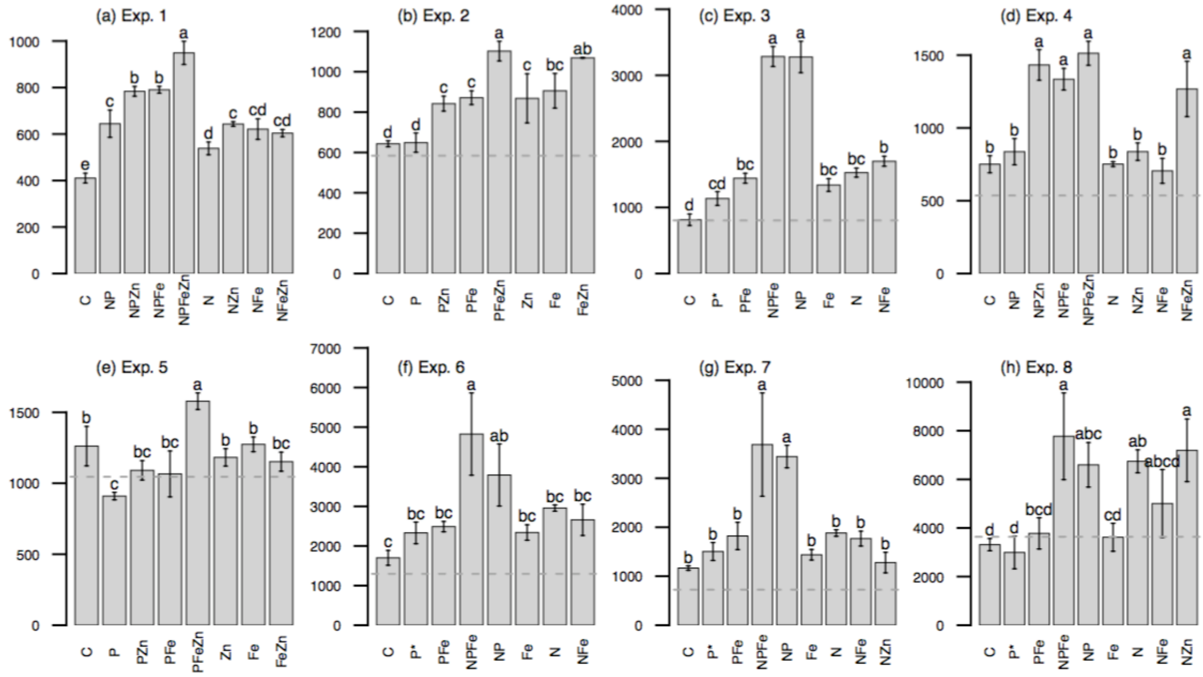

Supplementary Figure 3: Picoeukaryotic (~<2 μm) phytoplankton cell counts. Units: cells mL^{-1}. Shown are means ± standard errors, n=3 for all except where indicated by an asterisk (n=2). The grey horizontal line is the mean initial time point measurement (n=3 apart from Experiment 1, which has no cell counts for the initial time point). Treatment means were compared using a one-way ANOVA and a Fisher PLSD means comparison test (indistinguishable means labelled with the same letter (p<0.05); n.s. is 'not significant').

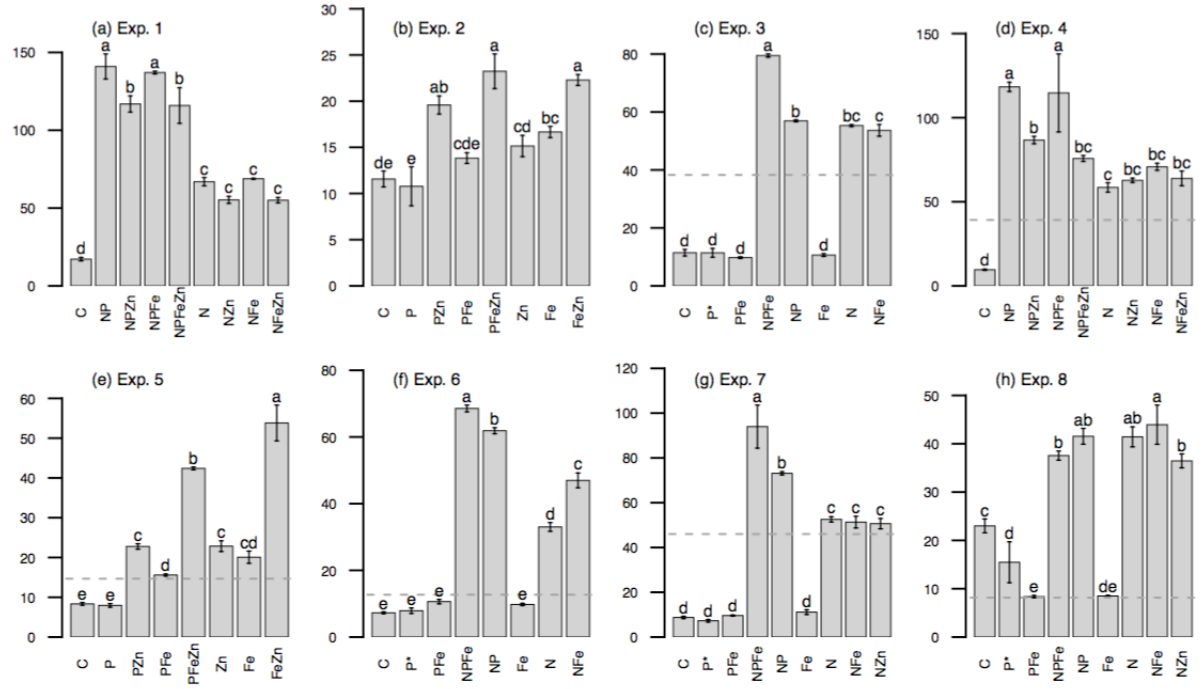

Supplementary Figure 4: *Synechococcus* chlorophyll-a fluorescence per cell. Arbitrary units. Shown are means ± standard errors, n=3 for all except where indicated by an asterisk (n=2). The grey horizontal line is the mean initial time point measurement (n=3 apart from Experiment 1, which has no cell counts/cellular fluorescence for the initial time point). Treatment means were compared using a one-way ANOVA and a Fisher PLSD means comparison test (indistinguishable means labelled with the same letter (p<0.05); n.s. is 'not significant').

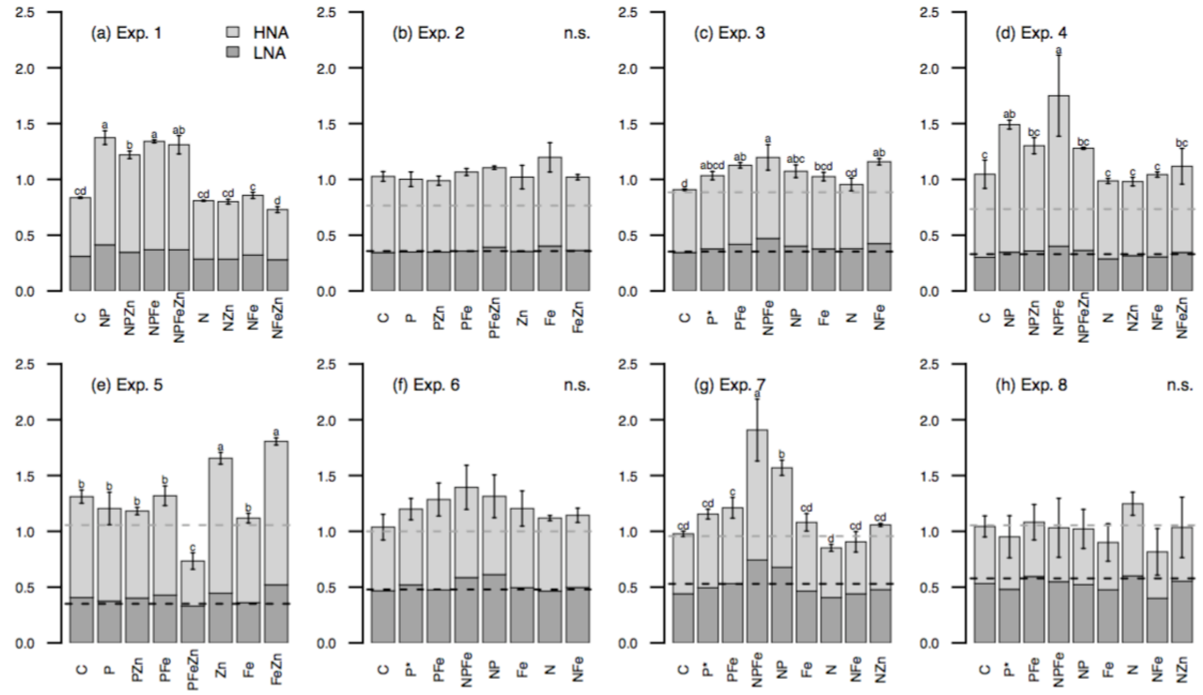

Supplementary Figure 5: Heterotrophic bacteria cell counts. Units: $\times 10^6$ cells mL^{-1}. LNA=low nucleic acid content; HNA=high nucleic acid content. Shown are means ± standard errors, n=3 for all except where indicated by an asterisk (n=2). Error bars are for LNA+HNA. The black/grey (LNA/HNA) horizontal lines are the mean initial time point measurements (n=3 apart from Experiment 1, which has no cell counts for the initial time point). Treatment means (LNA+HNA) were compared using a one-way ANOVA and a Fisher PLSD means comparison test (indistinguishable means labelled with the same letter (p<0.05); n.s. is 'not significant').

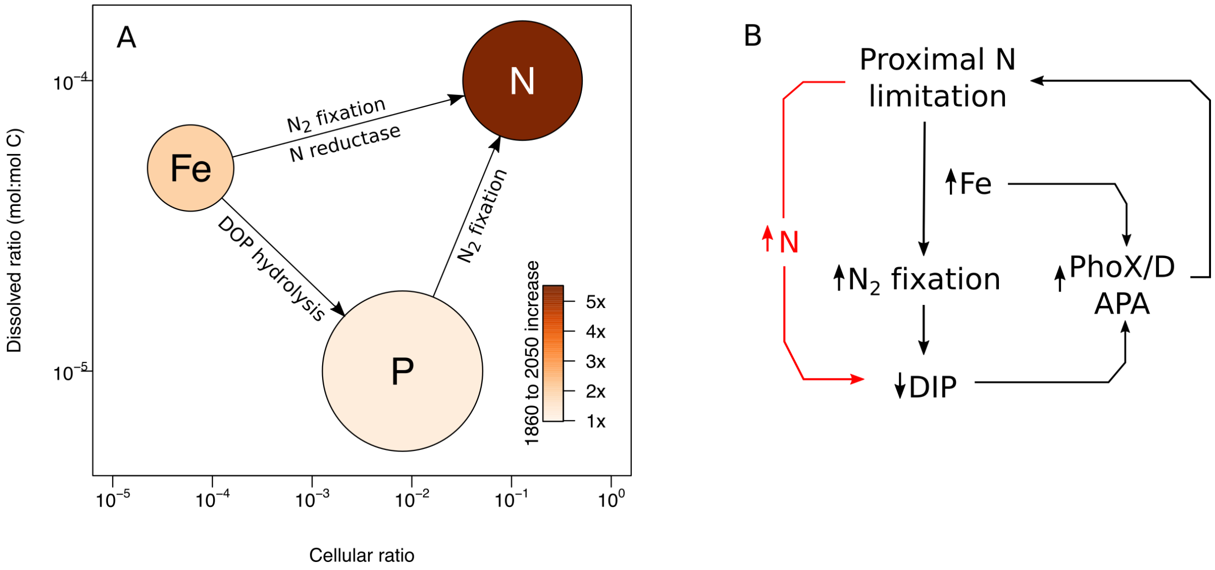

Supplementary Figure 6: Fe regulation of macronutrient acquisition pathways. (**a**) Control of bioavailable nutrient pools. In waters proximally limited by N, Fe availability dictates relative N_2 fixation via the Fe-dependant nitrogenase enzyme[4,5], although field evidence of P limitation of this process also exists[6,7]. For some species unable to access nitrate directly, Fe could also constrain N uptake via requirement in nitrate reductase[8]. Our findings imply a Fe-dependant bioavailable P pool. Arrows point in the direction of dependence on nutrient pool size and circle size represents the relative log-scaled oceanic residence time of the three elements, which span three orders of magnitude. Circle colours represent projected increases in aerosol-derived nutrients. Dissolved and cellular ratios, aerosol nutrient increases, and residence times are collated in Moore et al. (2013)[9], with ratios specific to subtropical North Atlantic seawater/organic material. (**b**) Nutrient limitation feedbacks. The modern, and likely the ancient, ocean is largely proximally N limited[9,10], and N_2 fixation is therefore favoured if there is a high Fe supply for nitrogenase cofactors. Enhanced growth via N input depletes DIP, which upregulates APA via PhoX/D that are activated by the available Fe. This process may play some role in ultimately maintaining proximal N limitation in low latitude oceans. Anthropogenic N input shortcuts N_2 fixation (red arrow), with biogeochemical impacts poorly resolved but could have a dependence on coincident fluxes of Fe.

Supplementary References

1. Luo, H., Benner, R., Long, R. & Hu, J. Subcellular localization of marine bacterial alkaline phosphatases. *Proc. Natl. Acad. Sci. U. S. A.* **106,** 21219–21223 (2009).
2. Dick, C. F., Dos-Santos, A. L. A. and Meyer-Fernandes, J. R. Inorganic phosphate as an important regulator of phosphatases. *Enzyme Res.*, 103980 (2011).
3. Mahaffey, C., Reynolds, S., Davis, C. E., Lohan, M. C. & Lomas, M. W. Alkaline phosphatase activity in the subtropical ocean: insights from nutrient, dust and trace metal addition experiments. *Front. Mar. Sci.* **1,** 1–13 (2014).
4. Berman-Frank, I., Cullen, J. T., Shaked, Y., Sherrell, R. M. & Falkowski, P. G. Iron availability, cellular iron quotas, and nitrogen fixation in *Trichodesmium*. *Limnol. Oceanogr.* **46,** 1249–1260 (2001).
5. Moore, C. M. *et al.* Large-scale distribution of Atlantic nitrogen fixation controlled by iron availability. *Nat. Geosci.* **2,** 867–871 (2009).
6. Sañudo-Wilhelmy, S. A. *et al.* Phosphorus limitation of nitrogen fixation by *Trichodesmium* in the central Atlantic Ocean. *Nature* **411,** 66–69 (2001).
7. Mills, M. M., Ridame, C., Davey, M., La Roche, J. & Geider, R. J. Iron and phosphorus co-limit nitrogen fixation in the eastern tropical North Atlantic. *Nature* **429,** 232–292 (2004).
8. Maldonado, M. T. & Price, N. M. Influence of N substrate on Fe requirements of marine centric diatoms. *Mar. Ecol. Prog. Ser.* **141,** 161–172 (1996).
9. Moore, C. M. *et al.* Processes and patterns of oceanic nutrient limitation. *Nat. Geosci.* **6,** 701–710 (2013).
10. Falkowski, P. G. Evolution of the nitrogen cycle and its influence on the biological sequestration of CO2 in the ocean. *Nature* **387,** 272–275 (1997).
